# Effect of pot design on the catch efficiency of snow crabs (*Chionoecetes opilio*) in the Barents Sea fishery

**DOI:** 10.1371/journal.pone.0219858

**Published:** 2019-07-18

**Authors:** Leonore Olsen, Bent Herrmann, Eduardo Grimaldo, Manu Sistiaga

**Affiliations:** 1 SINTEF Nord, Tromsø, Norway; 2 SINTEF Ocean, Trondheim, Norway; Department of Agriculture and Water Resources, AUSTRALIA

## Abstract

The snow crab (*Chioneocetes opilio*) fishery in the Barents Sea is carried out by large offshore vessels, as the fishing grounds are located far from shore and the gear must be transported back and forth over long distances. Therefore, fishers use stackable conical pots that allow large numbers of pots to be carried on deck for each trip. One of the drawbacks of using stackable pots is that the entrance is at the vertex of the conical pot, which fishers claim does not provide the desired fishing efficiency. Thus, the goal of this study was to determine whether a different pot design would improve the catch efficiency of snow crabs. We investigated the efficiency of a new type of pot called the moon pot, which provides continuous increased bait odour intensity as snow crabs make their way towards the entrance of the pot. This alteration was expected to increase catch efficiency compared to that of the conical pots used by the fleet today. However, experimental fishing results showed that the modified pots had significantly lower catch efficiency than the standard conical pots, as only ~66% of the number of crabs caught by the conical pots were caught in the moon pots. The main reason for this reduced catch efficiency likely was the initial steepness of the moon pot, which may have made it difficult for crabs to reach the pot entrance. These results demonstrated that pot design can dramatically affect catch efficiency of snow crabs.

## Introduction

The snow crab (*Chionoecetes opilio*) is a cold-water species that thrives at temperatures below 4 °C and typically inhabits Arctic regions. In the Barents Sea, the presence of snow crab was first recorded in 1996 [1], thus the commercial fishery is relatively new. In the period 2012–2015 the fishery grew very quickly, and Norwegian landings increased from 2500 kg in 2012 to 18,000 tonnes in 2015 with 11 Norwegian and 18 European Union vessels participating in the fishery [1]. However, in 2017 the landings were reduced to just over 3,000 tonnes because the Russian part of the Barents Sea, which is an area with a large population of snow crabs, was closed to the Norwegian and international fleets. In 2018, the snow crab quota in the Norwegian part of the Barents Sea was set at 4,000 tonnes, but the total catch was only 2,677 tonnes [2]. The decrease in landings experienced in the recent past has caused fishers to focus on exploring new areas in the Barents Sea and to increase the efficiency of the fishing gear.

The Norwegian snow crab fishery operates far off the coast and exclusively uses pots, thus only large factory vessels can participate in it. Depending on weather, catch, and ice conditions, each vessel deploys between 1,000 and 2,000 pots per day. The mesh size of the netting liner varies between 120 and 150 mm, but most vessels use 140 mm. A mesh size of 140 mm is believed to have an W50 (carapace width at which a crab has a 50% probability of being retained in the pot) of about 100 mm [3], which is the minimum legal size for snow crabs in the Barents Sea. The management regulations in the Norwegian fishery include a maximum of 12,000 pots deployed per vessel, a maximum soak time for pots of 3 weeks, a closed season (where fishers must remove all their pots) between June 15 and September 15, and a maximum of 20% of soft crabs in the catch. In addition, the minimum legal size of 100 mm carapace width means that in practice only male crabs can be landed (female snow crabs are rarely larger than 90 mm), and all undersized individuals must be returned to the sea. Norway regulates the snow crab fishery for the Svalbard Fisheries Protection Zone (International Council for the Exploration of the Sea (ICES) area SXV) and the Exclusive Economic Zone of Norway, which include large parts of the Barents Sea where this resource is targeted.

**Fig 1:**
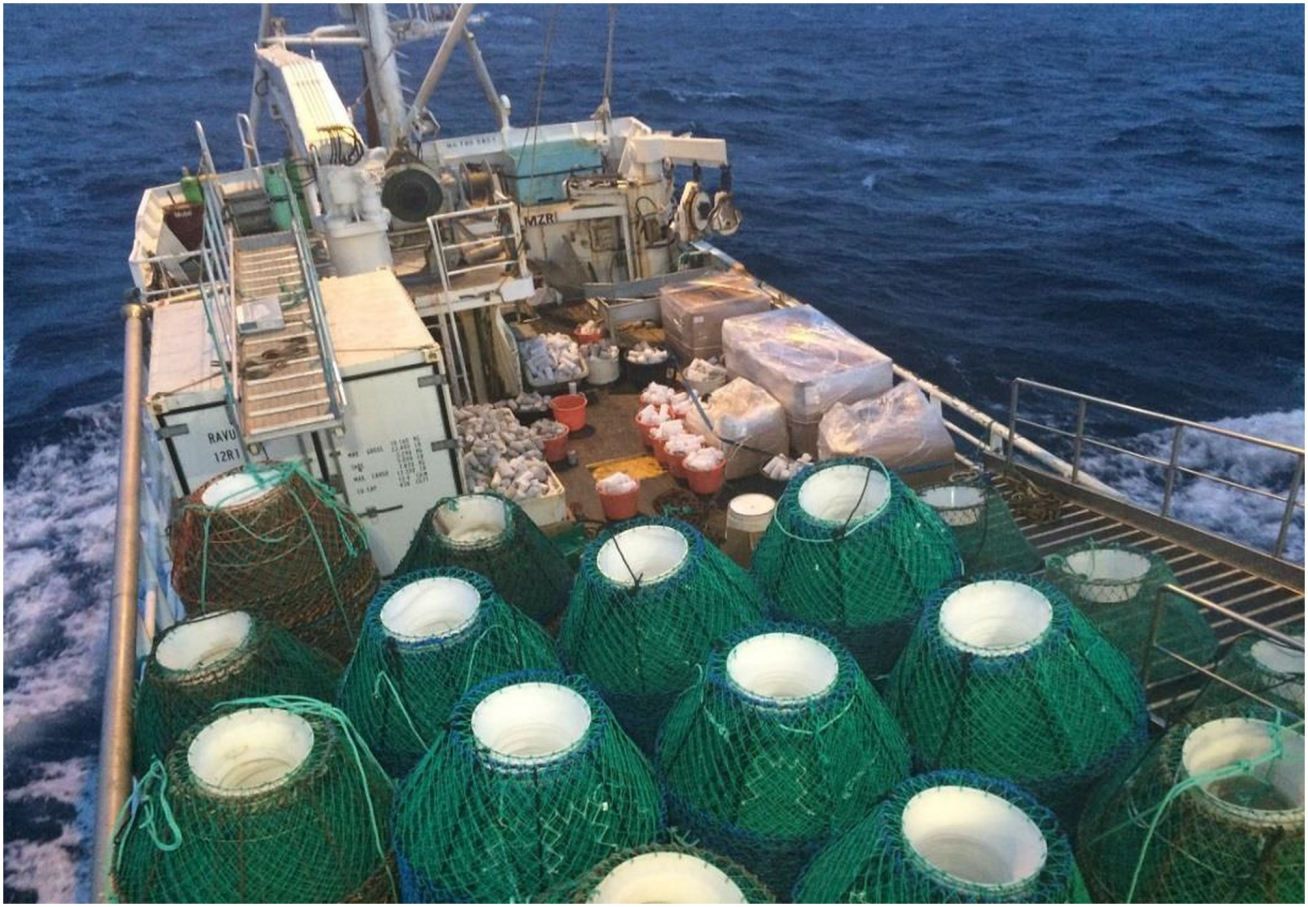
A Barents Sea snow crab vessel (MS North Eastern) on its way to the fishing grounds with the deck full of stacked conical pots (Photo: Opilio AS).

The standard conical pots used by the fishery were introduced from the Canadian east coast snow crab fishery to the Barents Sea snow crab fishery. The literature about design and catch efficiency of these pots is extensive [3–6]. These pots are light, manoeuvrable, and can be stacked, which is a major benefit when thousands of pots have to be transported from the coast to the fishing grounds (Fig 1). However, issues related to their catch efficiency in the Barents Sea fishery is a major concern to the fishing industry, and fishers have started looking for alternative designs to improve catch efficiency.

Bait position in the standard conical pot is one of the most important parameters affecting this type of pot’s catch efficiency. Vienneau et al. [7] reported that keeping the odour plume close to the seafloor (< 35 cm) and as close to the centre of the pot as possible was important to effectively attract snow crabs towards the pots and thereby increase the catch efficiency. However, the path that the crab must follow in order to enter the conical pot is not necessarily optimal with respect to the odour plume, as the pot’s entrance is at the vertex of the cone. Therefore, it is unclear whether or why a crab would continue climbing towards the top of a pot if by doing so it moved further away from the bait. In addition, the standard conical pot used today requires that a crab climb over an edge at the top of the pot before reaching the entrance. Whether this edge negatively influences the efficiency of the pots, either because it is difficult to climb or because at this point the crab is moving further from the bait, or both, remains unknown. Underwater recordings have shown that red king crabs (*Paralithodes camtschaticus*) were reluctant to climb over the edge on the top of the cone [8, 9]. The authors of these studies argued that this behaviour was a consequence of the orientation/distance of the crab relative to the bait odour plume. The same phenomenon may occur when snow crabs encounter conical pots.

In the present study, we compared the catch efficiency of a new pot design called the "moon pot" to that of the traditional conical pot used by most fishers in the Barents Sea today. We designed the present study to answer the following question: Is there any improvement in catch efficiency if moon pots are used instead of traditional conical pots?

## Materials and methods

### Ethics statement

All trials carried out during this study followed normal commercial fishing practice and the snow crab were not exposed to any additional harm. Therefore, this study did not require any permits from the authorities. Further, the trials did not involve any endangered or protected species.

### Pot designs

**Fig 2:**
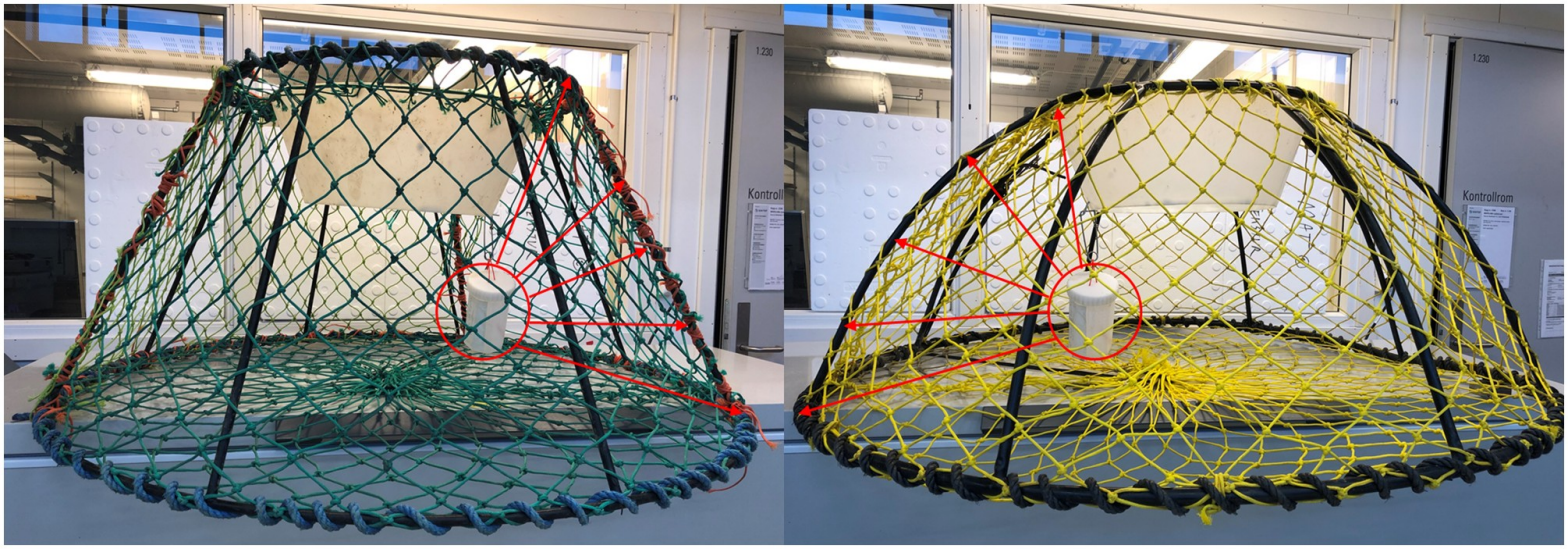
Comparison of the geometry and dimensions of the standard conical pots (control pots) and moon pots (experimental pots). The arrows illustrate the distances from the pot’s wall to the bait container.

Standard conical pots used by the snow crab fishery along the east coast of Canada [6] were used as control pots. Each conical pot had a diameter of 70 cm at the top and 130 cm at the bottom, was 60 cm high, and had an entrance made of a hard-plastic funnel with an outer diameter of 55 cm (Fig 2). The frame was made of 12-mm steel bars and the lower ring was made of a 14-mm steel bar, yielding a pot weight of approximately 12.5 kg. The netting liner was made of Ø4 mm single polyethylene (PE) twine and the nominal mesh size was 140 mm.

**Fig 3:**
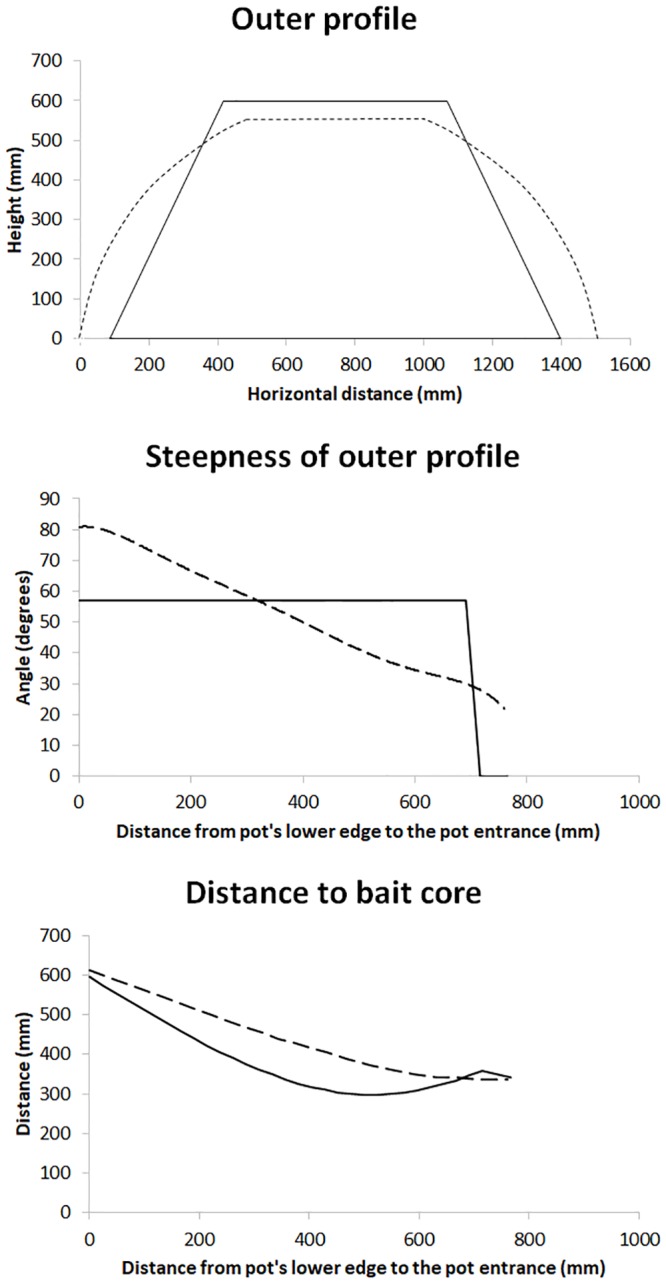
Geometry differences between the pots. (a) Outer profile of the standard conical pot (solid line) and the moon pot (dashed line). (b) Steepness of the outer profile along the path to the pot entrance for each of the pots. (c) Distance to the bait core along the path to the pot entrance for each of the pots.

We used a new type of pot called the moon pot as the experimental gear (Fig 2). The moon pot had a diameter of 55 cm at the top, a base with a diameter of 145 cm, and a maximum height of 57 cm. The hard-plastic funnel at the entrance of this pot was identical in size and shape to that used in the conical pot. The netting liner was made of Ø4 mm single PE twine and the nominal mesh size was 140 mm. The moon pot frame was made of 12-mm steel bars and the lower ring was made of 14-mm steel bars, yielding a pot weight of approximately 14 kg. The moon pot was 3 cm shorter but had a wider base (15 cm greater diameter) than the standard conical pot (Fig 3a). The moon pot’s wall also had a steep initial angle of approximately 80 degrees that gradually decreased to approximately 22 degrees at the pot entrance; in contrast, the conical pot had a constant angle of approximately 63 degrees from the base to the upper edge of the pot (Fig 3b). The relative distance to the bait core along the pot’s wall that a snow crab must follow to enter the pot is different between standard conical pots and moon pots. In the moon pot, this distance continuously gets smaller as a snow crab approaches the pot entrance (Fig 3c).

Because mesh size and shape can influence the relative catch efficiency of a pot, width and height of all meshes in a typical pot of both types were measured. Subsequently, mesh size, opening mesh angle, and hanging ratio were calculated based on simple geometrical relations for each mesh in the pots. We used an unpaired *t*-test with unequal variances to test for significant differences in mesh size, mesh opening angle, and hanging ration between the two types of pots. The *F*-test was used to assess significant differences in the variances. Due to the outer profile of the pots (Fig 3a) mesh shape differed between horizontal mesh rows. Therefore, the tests described above was carried out for individual mesh rows separately.

### Data collection

Sea trials were carried out on-board the fishing vessel “North Guider” (55.2 m overall length and 2250 HP) in the central Barents Sea (N76°28.9–E36°36.9 and N75°56.1–E37°33.8 (ICES area SXV)) at depths of 240–310 m. The trials took place between March 1 and April 9 of 2018. The pots were fished on longlines with 200–400 pots attached to the mainline every 30 m by a quick link system that allowed quick attachment and release of the pots to/from the mainline. The number of pots per longline depended on the ability to set longlines in an area given other deployed longlines. Standard and test pots were deployed on the same longline. After every second or third control pot there was a test pot. Each pot was baited with approximately 700 g of squid (*Illex spp*.). When the pots were hauled on board, they were emptied separately onto a sorting board where the carapace width of each crab was measured to the nearest mm using a calliper. Subsampling was avoided.

### Measure to quantify the effect of pot design on catch efficiency

The estimation of the absolute catch efficiency for the standard conical pot and the new moon pot requires catch data from fishing the pots and data on the numbers and sizes of snow crab available on the fishing grounds at the time and place the pots are being fished. However, the latter information is not available because it cannot be obtained with the experimental design applied in this study. But the alternative of just modelling and estimating the total catch or catch per unit effort for each pot type would have had very limited general value because it depends on the availability of crab, which vary with time and fishing ground. Further, the absolute catch for each pot type depends on soak time in an unknown way. Specially, the capture process in pots involves an attraction/entry process and a subsequent but overlapping size selection process, where some of the crab that have entered a pot can escape. Both these processes are affected by the intensity of the odour of the specific bait type used, which will change over time. Therefore, assuming for example that the total catch increases linearly with increasing soak time is a very questionable assumption to apply. Further, as the capture efficiency may likely be different for different sizes of snow crab, the estimation of absolute catch efficiency should explicitly include potential size-dependency which again, would need detailed information on the size-dependent availability of crab.

The considerations described above show that estimating the absolute catch efficiency for the two types of pots fished in the experiments is very difficult and that only estimating their respective total catches would provide very little information of general interest. However, the estimation of the effect of changing pot design on the size-dependent catch efficiency for crab does not require an estimation of the absolute catch efficiency for each pot type because the effect can be quantified by the relative catch efficiency between the two pot types in terms of a ratio between catches. Contrary to the absolute size-dependent catch efficiency, the relative catch efficiency between the two pot types can be estimated based on catch data from the experimental fishing and does not require information on the size-dependent availability of snow crab on the fishing ground. To use this approach the two types of pot need to be deployed simultaneously on the same fishing grounds with same deployment pattern regarding soak time and baiting. Further, this approach can avoid having to rely on a model relating capture and soak time. As the relative catch efficiency between the pot types does not depend on the specific availability of snow crab, this result should be of much more general interest than results that highly depend on specific and varying crab availability. Therefore, this study uses the relative size-dependent catch efficiency between the two pot types to quantify the effect on catch efficiency by changing pot design.

The relative size-dependent catch efficiency between two fishing gears deployed simultaneously is often referred as catch ratio in literature. Well-established methods exist for estimating catch ratio between two gears for both active [10–24] and passive fishing gears [25–32], including for pots [25]. In this study, we applied the method described in the following section to estimate the catch ratio between the two types of snow crab pots.

### Estimation of the size-dependent catch ratio between standard conical pots and new moon pots

This section describes in detail how the size-dependent catch ratio analysis was carried out based on the collected data to estimate the relative catch efficiency between standard and moon pots.

Because the standard and moon pots were fished simultaneously on the same longlines, the total catches between the two pot types for each longline deployment could be compared as pairs. However, each longline covered a long track (approx. 6500 m) which could lead to variation in snow crab population available to be caught for pots placed at different locations along the longline. Therefore, for analysis purposes the longline was segmented into local groups (sets). In this way it was more realistic to assume that the standard and the test pots were fishing on the same population of snow crabs than if each set were covering the complete longline. Therefore, the total catch between the two pots types was paired for each set. A set consisted of between 35 and 72 standard pots and 38 and 73 test pots. The approach of comparing the total catches between two types of pots, and the sum of the pots in the set, was first applied by Xu and Millar [33]. In their case, each set consisted of four experimental test pots and two control pots. However, in our case more pots were attached to each longline and the catch level per pot was much smaller, thus, favouring using higher numbers of pots for each set.

We used the statistical analysis software SELNET [30, 34] to analyse catch data and to conduct size-dependent catch comparisons and catch ratio analyses. The size was represented by the carapace width, *w*, for each of the snow crabs caught. Using the numbers and sizes of snow crabs caught in test and standard pots in each set, we determined whether there was a significant difference in the catch efficiency averaged over deployments between the test pot and standard pot types. Specifically, we assessed the relative length-dependent catch efficiency effect of changing from standard pots to moon pots using the method described in Herrmann et al. [31] and compared the catch data for the standard and the test pots. This method models the size-dependent catch comparison rate (*CC*_*w*_) summed over sets:

$${CC}_{w}=\frac{\sum_{j=1}^{m}\left\{\frac{{nt}_{wj}}{{qt}_{j}}\right\}}{\sum_{j=1}^{m}\left\{\frac{{nt}_{wj}}{{qt}_{j}}+\frac{{ns}_{wj}}{{qs}_{j}}\right\}}$$ (1)

where *ns*_*wj*_ and *nt*_*wj*_ are the numbers of snow crabs caught in each size class *w* in a standard pot and a test pot in set *j*. *m* is the number of sets. *qs*_*j*_ and *qt*_*j*_ are sampling factors introduced to account for unequal numbers of standard (*cs*_*j*_) and test (*ct*_*j*_) pots fished in the individual sets *j*. Specifically, *qs*_*j*_ and *qt*_*j*_ were set at:

$$\begin{array}{c} {qs}_{j}=\frac{{cs}_{j}}{max({cs}_{j},{ct}_{j})}\\ {qt}_{j}=\frac{{ct}_{j}}{max({cs}_{j},{ct}_{j})} \end{array}$$ (2)

The functional form for the catch comparison rate *CC(w*, *v)* was obtained using maximum likelihood estimation by minimizing the following expression:

$$-\sum_{w}\left\{\sum_{j=1}^{m}\left\{\frac{{nt}_{wj}}{{qt}_{j}}\times ln(CC(w,v))+\frac{{ns}_{wj}}{{qs}_{j}}\times ln(1.0-CC(w,v))\right\}\right\}$$ (3)

where ***v*** represents the parameters describing the catch comparison curve defined by *CC(w*, *v)*. The outer summation in expression (3) is the summation over size classes *w*. When the catch efficiency of the moon and standard pots is similar, the expected value for the summed catch comparison rate is 0.5. Therefore, this baseline can be applied to judge whether there is a difference in catch efficiency between the two types of pots. The experimental *CC*_*w*_ was modelled by the function *CC(w*, *v)* as follows:

$$CC(w,v)=\frac{exp(f(w,v_{0},\ldots ,v_{k}))}{1+exp(f(w,v_{0},\ldots ,v_{k}))}$$ (4)

where *f* is a polynomial of order *k* with coefficients *v*_*0*_ to *v*_*k*_. The values of the parameters ***v*** describing *CC(w*, *v)* were estimated by minimizing expression (3), which was equivalent to maximizing the likelihood of the observed catch data. We considered *f* of up to an order of 4 with parameters *v*_*0*_, *v*_*1*_, *v*_*2*_, *v*_*3*_, and *v*_*4*_. Leaving out one or more of the parameters *v*_*0*_*… v*_*4*_ led to 31 additional models that were also considered as potential models for the catch comparison *CC(w*, *v)*. Among these models, estimations of the catch comparison rate were made using multi-model inference to obtain a combined model [30, 35].

The ability of the combined model to describe the experimental data was evaluated based on the *p*-value. This *p*-value, which was calculated based on the model deviance and the degrees of freedom, should not be < 0.05 for the combined model to describe the experimental data sufficiently well, except for cases in which the data exhibited over-dispersion [30, 36]. Based on the estimated catch comparison function *CC(w*, *v)* we obtained the relative catch efficiency (also called the catch ratio, *CR(w*, *v)*) between the two pot types based on the following relationship:

$$CR(w,v)=\frac{CC(w,v)}{\left(1-CC(w,v)\right)}$$ (5)

The catch ratio is a value that represents the ratio between catch efficiency of test and standard pots. Thus, if the catch efficiency of both pot types is equal *CR(w*,*v)* should be 1.0. Thus, *CR(w*, *v)* = 1.5 would mean that the test pots are catching 50% more snow crabs with size *w* than the standard pots. In contrast, *CR(w*, *v)* = 0.7 would mean that the test pots are catching only 70% of snow crabs with size *w* that the standard pots are catching.

The confidence intervals for the catch comparison curve and catch ratio curve were estimated using a double bootstrapping method [31, 34]. This bootstrapping method accounts for between-set variability (the uncertainty in the estimation resulting from set variation of catch efficiency in the pots and in the availability of snow crabs) as well as within-set variability (uncertainty about the size structure of the catch for the individual sets). However, contrary to in Sistiaga et al. [21], but similar to Herrmann et al. [34], the outer bootstrapping loop in the current study that accounted for the between-deployment variation was performed paired for the test and standard pots, taking full advantage of the experimental design in which both types of pots were deployed simultaneously in the individual sets. By multi-model inference in each bootstrap iteration, the method also accounts for the uncertainty in model selection. We performed 1,000 bootstrap repetitions and calculated the Efron 95% confidence intervals [37]. To identify sizes of snow crabs with significant differences in catch efficiency, we checked for size classes for which the 95% confidence intervals for the catch ratio curve did not contain 1.0.

Size-integrated average values for the catch ratio (*CR*_*average*_) were estimated directly from the experimental catch data using the following equations:

$$\begin{array}{c} {CR}_{average-}=\frac{\sum_{w<mw}\sum_{j=1}^{m}\left\{\frac{{nt}_{wj}}{{qt}_{j}}\right\}}{\sum_{w<mw}\sum_{j=1}^{m}\left\{\frac{{ns}_{wj}}{{qs}_{j}}\right\}}\\ {CR}_{average+}=\frac{\sum_{w\geq mw}\sum_{j=1}^{m}\left\{\frac{{nt}_{wj}}{{qt}_{j}}\right\}}{\sum_{w\geq mw}\sum_{j=1}^{m}\left\{\frac{{ns}_{wj}}{{qs}_{j}}\right\}} \end{array}$$ (6)

where the outer summations include the size classes in the catch during the experimental fishing period that were under (for *CR*_*average*−_) and over (for *CR*_*average*+_) the minimum targeted size (*mw* = 100 mm carapace width) of snow crabs. In contrast to the size-dependent evaluation of the catch ratio *CR(w*, *v)*, *CR*_*average*−_ and *CR*_*average*+_ are specific for the population structure encountered during the experimental trials. Therefore, those values are specific for the size structure in the fishery at the time the trials were carried out, and it cannot be extrapolated to other scenarios in which the size structure of the snow crab population may be different unless it should turn out that the catch ratio between the two types of pots show no dependency of crab size.

Finally, to investigate how well the size selectivity of the test and standard pots matched the size structure of snow crabs in the area fished, two fishing sustainability indicators (*NRatio*) were estimated directly from the experimental catch data by:

$$\begin{array}{c} {NRatio}_{Test}=\frac{\sum_{w<mw}\sum_{j=1}^{m}\left\{\frac{{nt}_{wj}}{{qt}_{j}}\right\}}{\sum_{w\geq mw}\sum_{j=1}^{m}\left\{\frac{{nt}_{wj}}{{qt}_{j}}\right\}}\\ {NRatio}_{Standard}=\frac{\sum_{w<mw}\sum_{j=1}^{m}\left\{\frac{{ns}_{wj}}{{qs}_{j}}\right\}}{\sum_{w\geq mw}\sum_{j=1}^{m}\left\{\frac{{ns}_{wj}}{{qs}_{j}}\right\}} \end{array}$$ (7)

where the outer summations include the size classes in the catch during the experimental fishing period that were under (in the nominator) and over (in the denominator) the minimum targeted size of snow crabs. *NRatio* quantifies the ratio between undersized and target sizes of snow crabs captured. Ideally, *NRatio* should be as low as possible. The value of *NRatio* is affected by both the size selectivity of the pot and the size structure of the crabs in the fishing grounds. Therefore, it provided an estimate that is specific for the population fished and it could not be extrapolated to other areas and seasons.

## Results

### Mesh size, mesh opening angle, and hanging ratio

No significant difference in mean mesh size between the two pot types was detected for any of the six mesh rows nearest the bottom of the pots (Table 1; *t*-test, *p* > 0.05). The mean mesh size for the lowest three rows, which likely are most important for pot escapement [6], ranged from 125.9 to 129.7 mm for the conical pot and from 127.0 to 128.1 mm for the moon pot. Thus, the mesh sizes recorded for both pot types were smaller than the size that is expected to enable all undersized crabs to escape (137 mm) [38]. Mesh opening angle and hanging ratio also potentially can affect which sizes of snow crabs are able to escape through a diamond mesh [38]. For the first mesh row, the moon pot had a significantly lower mean opening angle and hanging ratio than the conical pot (Table 1; *t*-test, *p* < 0.001). However, this was not the case for the next four mesh rows. The conical pots had significant higher variance in mesh size than the moon pot (Table 1; *F*-test, *p* < 0.05).

**Table 1:** Mean (SD) mesh opening size, mesh opening angle, and hanging ratio for both types of pots and significance of statistical tests. Significant values are given in bold.

Mesh row #	1	2	3	4	5	6
Conical pot						
Number of meshes	33	31	30	28	26	25
Mesh size (mm)	125.9 (4.5)	126.2 (6.3)	129.7 (7.0)	130.8 (7.1)	129.4 (4.4)	130.5 (6.2)
Mesh opening angle (degrees)	61.6 (9.4)	67.9 (7.5)	74.4 (5.4)	79.9 (5.6)	88.3 (7.3)	95.9 (8.2)
Mesh hanging ratio	0.81 (0.06)	0.78 (0.05)	0.73 (0.04)	0.70 (0.04)	0.63 (0.05)	0.57 (0.07)
Moon pot						
Number of meshes	36	36	35	35	35	34
Mesh size (mm)	127.2 (2.8)	128.1 (1.9)	127.0 (1.6)	129.3 (2.9)	128.3 (3.9)	127.6 (2.0)
Mesh opening angle (degrees)	52.0 (6.8)	65.5 (10.8)	72.2 (4.6)	82.9 (5.8)	92.4 (7.6)	108.1 (4.7)
Mesh hanging ratio	0.87 (0.03)	0.79 (0.07)	0.75 (0.03)	0.67 (0.04)	0.60 (0.06)	0.47 (0.04)
p-value *t*-test between pot types						
Mean mesh size	0.164	0.124	0.054	0.321	0.290	0.064
Mean mesh opening angle	**< 0.001**	0.279	0.089	0.055	0.059	**< 0.001**
Mean mesh hanging ratio	**< 0.001**	0.363	0.085	0.449	0.319	**< 0.001**
p-value *F*-test between pot types						
Variance mesh size	**0.009**	**< 0.001**	**< 0.001**	**< 0.001**	0.515	**< 0.001**
Variance mesh opening angle	0.061	**0.047**	0.364	0.805	0.849	**0.003**
Variance mesh hanging ratio	**0.003**	**0.073**	0.234	0.630	0.709	**0.010**

### Data collected

A total of 18 sets of pots each containing between 35 and 72 standard pots and between 39 and 73 test pots were included in the analysis, which equalled a total of 835 standard conical pots and 851 moon pots (Table 2). During the sea trials, the number of crabs caught in the standard and test pots varied between sets from 40 to 129 and between 20 and 103, respectively. Between sets the mean number of crabs (± SD) caught per standard pot varied between 0.76 (± 0.99) and 2.63 (± 1.84) and for the moon pot between 0.48 (± 0.67) and 2.29 (± 2.15). Although soaking time varied between 5 and 16 days, it was not accounted for in the analysis because it equally affected both the test and standard pots paired in the individual sets. In total 1,435 snow crabs were caught in the standard pots, and 975 were caught in the moon pots, respectively (Table 2). Thus, 2,410 snow crabs were included in the study.

**Table 2:** Experimental data set. Individual sets of pots with corresponding start position, depth, soaking time, number of pots used, and the number of snow crabs caught in the different pots used in the analysis.

Long line	Set ID	Latitude	Longitude	Depth (m)	Soaking time (days)	Number of standard pots	Snow crabs in standard pots	Mean (±SD) number of crabs per standard pots	Number of moon pots	Snow crabs in moon pots	Mean (±SD) number of crabs per moon pot
1	1	76°08'22"N	37°22'30"E	240	7	49	76	1.55 (± 1.69)	47	29	0.62 (± 0.82)
1	2	76°08'22"N	37°22'30"E	240	7	39	90	2.31 (± 1.67)	53	89	1.68 (± 1.92)
1	3	76°08'22"N	37°22'30"E	240	7	39	43	1.10 (± 1.33)	49	38	0.76 (± 1.36)
2	4	76°07'58"N	37°20'36"E	261	6	72	55	0.76 (± 0.99)	41	20	0.53 (± 0.82)
2	5	76°07'58"N	37°20'36"E	261	6	42	53	1.26 (± 1.25)	73	78	1.07 (± 1.66)
2	6	76°07'58"N	37°20'36"E	261	6	57	129	2.26 (± 1.99)	60	103	1.72 (± 1.98)
3	7	76°15'06"N	37°19'26"E	254	10	49	70	1.40 (± 1.51)	46	76	1.65 (± 1.64)
3	8	76°15'06"N	37°19'26"E	254	10	43	72	1.67 (± 1.41)	45	31	0.68 (± 0.95)
4	9	76°18'09"N	36°41'14"E	249	7	43	73	1.69 (± 1.98)	39	43	1.10 (± 1.23)
4	10	76°18'09"N	36°41'14"E	249	7	45	93	2.07 (± 1.76)	44	65	1.48 (± 1.50)
5	11	76°12'54"N	35°39'42"E	296	16	48	100	2.08 (± 1.41)	47	60	1.28 (± 1.25)
5	12	76°12'54"N	35°39'42"E	296	16	42	99	2.36 (± 2.02)	42	58	1.38 (± 1.99)
6	13	76°05'59"N	37°01'24"E	265	7	35	40	1.14 (± 0.97)	52	25	0.48 (± 0.67)
6	14	76°05'59"N	37°01'24"E	265	7	41	93	2.27 (± 1.83)	42	43	1.02 (± 1.29)
7	15	76°08'51"N	35°54'57"E	274	11	60	121	2.01 (± 1.79)	44	55	1.25 (± 1.57)
7	16	76°08'51"N	35°54'57"E	274	11	47	42	0.89 (± 1.27)	45	25	0.56 (± 0.89)
8	17	76°05'00"N	35°44'58"E	287	5	43	78	1.86 (± 1.35)	44	50	1.14 (± 1.09)
8	18	76°05'00"N	35°44'58"E	287	5	41	108	2.63 (± 1.84)	38	87	2.29 (± 2.15)

### Catch efficiency of moon pots versus standard conical pots

**Fig 4:**
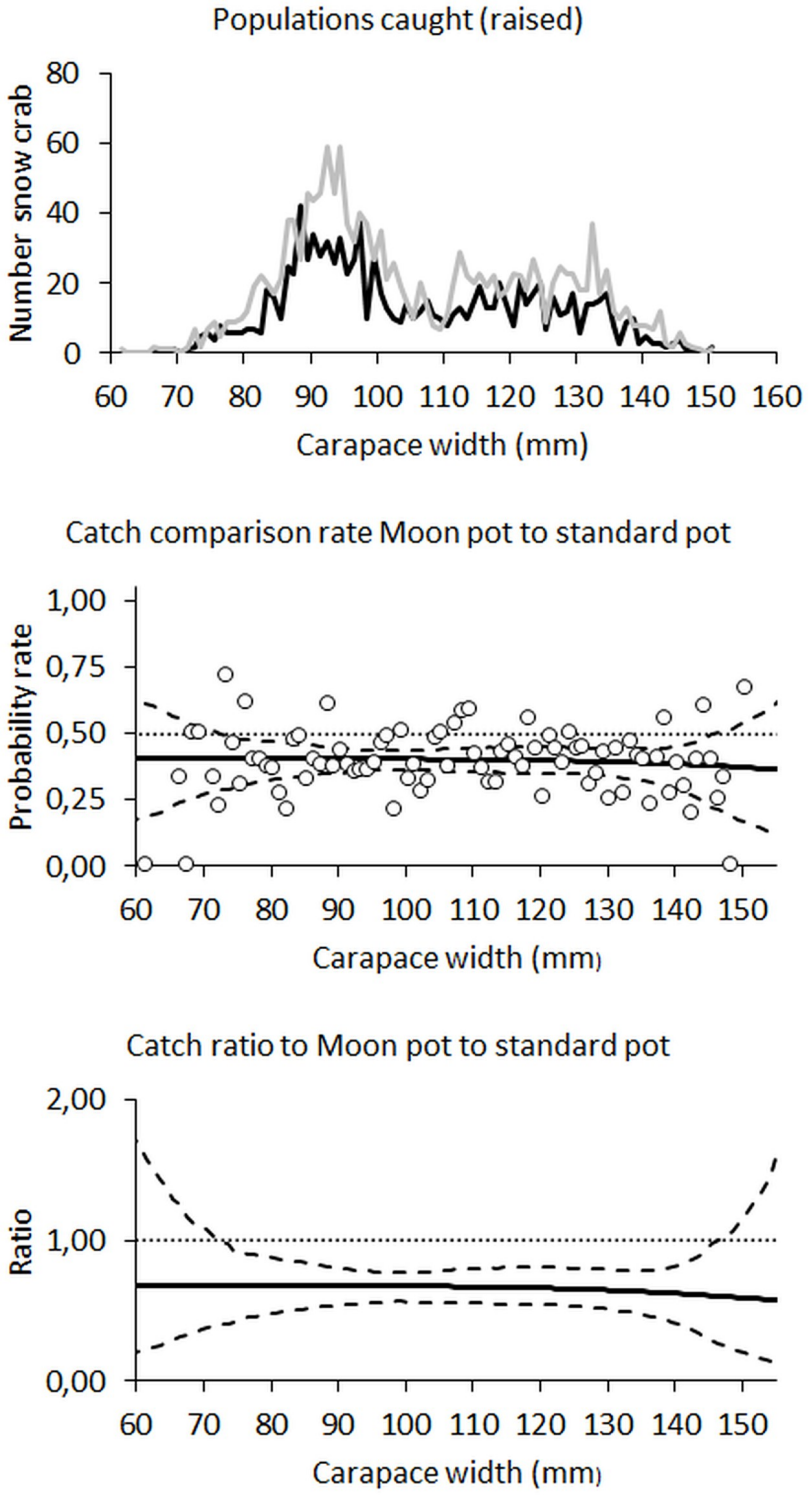
Populations caught (first row), catch comparison rates (centre row), and catch ratios (bottom row) for standard pots against moon pots. In the population plots the black curve represents the population caught in the test pots, and the grey curve represents the raised population caught in the standard pots. The experimental catch comparison rate [according to Eq (1)] is represented by circles. For the modelled catch comparison rate and catch ratio, the full black curve represents the estimated mean curve and the stippled curves represent the 95% confidence intervals. Horizontal dotted curves represent baseline for no effect of change in pot design on catch comparison rate (at 0.5) and catch ratio (at 1.0), respectively.

In general, the modelled catch comparison curve followed the main trend found in the experimental data (Fig 4). This is collaborated by the p-value at 0.226 showing that the deviation between the experimental catch comparison rate and the modelled curve well could be a coincidence (Table 3). Therefore, we were confident in using the modelled catch comparison curve and associated catch ratio curve to assess the effect on catch efficiency of switching from the standard conical pot to the moon pots. Both curves were nearly horizontal, implying only a very weak snow crab size dependency on the relative catch efficiency between the two types of pots (Fig 4, Table 3).

**Table 3:** Catch ratio (*CR(w)*) (%) and fit statistics obtained for the moon pots versus the standard pots. *CR(w)* values that are significantly different from 100% (i.e., those with same fishing efficiency as standard pots) are in bold. Values in brackets represent 95% confidence intervals. DF = degrees of freedom.

w (mm)	*CR(w)* (%) to standard pot
70	67.54 (37.76–107.55)
75	**67.57 (44.31–92.30)**
80	**67.61 (48.65–87.90)**
85	**67.65 (51.93–83.93)**
90	**67.68 (54.21–80.29)**
95	**67.66 (56.17–78.02)**
100	**67.59 (56.38–77.55)**
105	**67.44 (55.93–78.61)**
110	**67.20 (55.61–80.31)**
115	**66.83 (54.53–81.20)**
120	**66.33 (54.38–81.55)**
125	**65.65 (53.40–80.54)**
130	**64.80 (51.14–79.28)**
135	**63.74 (46.89–79.12)**
140	**62.47 (40.48–83.27)**
145	**60.98 (28.84–98.22)**
150	59.29 (19.71–118.81)
*CR*_*average*−_	**67.33 (53.49–83.23)**
*CR*_*average*+_	**65.95 (53.68–79.58)**
*NRatio*_*Test*_	0.97 (0.79–1.33)
*NRatio*_*Standard*_	0.95 (0.73–1.20)
*p*–value	0.2260
Deviance	88.12
DF	79

The length distribution of snow crabs showed that both type of pots caught a large proportion of individuals below the minimum target size (< 100 mm carapace width) (Fig 4, first row). This was corroborated by the estimated values for *NRatio*, which indicated capture of approximately one undersized crab for each of the targeted sizes (Table 3). These results confirmed that mesh sizes (Table 1) should be bigger to enable all undersized crabs to escape [38].

The catching efficiency of the moon pots was lower than that of the standard pots (Fig 4, Table 3). Over a wide size range the relative catch efficiency of the moon pots was only about 62–68% of that of the standard conical pot, and the upper confidence limit was below 85% for several sizes of snow crabs. These results, averaged over the sizes of snow crabs below and above 100 mm carapace width, showed that the moon pots caught, respectively, 67.33% and 66.95% of what the standard pots caught (*CR*_*average*−_ and *CR*_*average*+_ in Table 3). The weak size dependency in the relative catch efficiency between the moon pots and the standard pots imply that the values can to an extent be extrapolated to other population scenarios (Fig 4).

## Discussion

In the present study we investigated the catch efficiency of a new type of pot (the moon pot) and compared it to that of standard conical pots used by the snow crab fleet fishing in the Barents Sea. The long-term goal was to design a pot to improve the low catch rates of standard conical pots in this fishery. We speculated that bait position within the pots influenced their catch efficiency. In a standard conical pot, the shortest distance to the bait, and consequently the position that creates the strongest odour plume, occurred when bait was placed in the middle of the pot’s wall (Figs 2 and 3). However, this placement was not ideal because a snow crab likely would not follow a path in which the bait smell did not continuously increase, and it would not be motivated to climb all the way to the pot entrance at the top of the pot if the odour plume at the top of the pot was weaker than it is in the middle of the pot. Stiansen et al. [8, 9] showed how red king crabs interacted with baited pots and how crabs turned back after reaching the conical pot’s upper edge, thus not reaching the pot opening and consequently not getting caught. On average, one king crab was caught for every 30 climb attempts. Therefore, in the new pot design we tried to reduce the relative distance between the crabs and the bait source as the crabs climb the pot towards the pot entrance.

Our results showed clear differences in catch efficiency between the standard pots and moon pots (Table 2). Exchanging the standard conical pot for the moon pot resulted in around 34% reduction of the snow crab catch (Table 3). One possible explanation for the low efficiency of moon pots was that the negative effect of the steep side wall angle at the lowest part of the moon pot outweighed the positive effect of a path with a continuous increase in bait smell. We speculated that the challenge of climbing the steep wall (up to 80 degrees) to reach the pot entrance of the moon pot was too big.

A large proportion of snow crabs caught in the pots in this study were below the minimum target size (100 mm carapace width). Whether this result was due to a great abundance of undersized crabs in the area and/or the lack of size selection in the pots was unknown. However, the mesh size used was smaller than that recommended in previous studies [38, 39], which may have contributed to the retention of many undersized crabs. Researchers have reported that pots need at least 14 days for all undersized snow crabs (< 100 mm carapace length) to exit the pot to reach 100% size selection at the seabed [38, 39]. Although soaking time varied between 5 and 16 days in our study and may have affected size selection, it was not accounted for in the analysis because it equally affected both the test and the standard pots being paired in the individual sets.

Even though the new pot design did not provide the desired increase in catch efficiency, this study demonstrated that catch efficiency can be dramatically affected by small changes in pot design. We believe that the position of the bait, the position of the odour plume with respect to the position of the pot entrance, and the pot shape are key factors that influence the catch efficiency of pots. Previous experiments showed that side entrance pots caught up to three times more king crabs than conical top entrance pots, but the difference decreased with increasing soak time [8, 9]. Therefore, the challenge now is to design stackable side-entrance pots, as a snow crab vessel in the Barents Sea carries up to 12,000 pots per trip, whereas a red king crab vessel carries only up to 120 pots.

## Supporting information

S1 Fig Catch data for individual sets. The catch data consist of count data for number of snow crab caught in respectively the moon pots (Test 1) and standard conical pots (Test 2) for each size class (Length) corresponding to the carapace width.
(ZIP)
